# Result of the Last Hundred Cases of Labour

**Published:** 1829-05

**Authors:** 

**Affiliations:** his Pupils


### MIDWIFERY.

*Result of the last Hundred Cases of Labour, attended by* Mr. Jewel *and his Pupils; as stated in a Lecture to his Class.*

| | |
|---|---|
| Natural . . . . . . . . . . | 73 |
| Premature . . . . . . . . . | 1 |
| Protracted beyond twenty-four hours | 14 |
| Twins . . . . . . . . . . . . | 2 |
| Feet presentation . . . . . . | 2 |
| Breech ditto . . . . . . . . | 4 |
| Arm ditto . . . . . . . . . . | 1 |
| Hemorrhage after delivery . . . | 2 |
| Forceps case . . . . . . . . . | 1 |
| Total . . . . . | 100 |

Mr. Jewel observed, that he copied this statement from a book, which contained a faithful record of all cases of labour attended by himself and pupils in private and dispensary practice, and which were arranged under the following heads: *Name—residence—when delivered—sex of infant—case—observations.*

He had brought this statement before his class more as a matter of interest and curiosity to the students individually, than as a criterion by which they were to judge of the frequency of difficult or unusual cases. Calculations of that kind had already, and with a great degree of accuracy, been made from the registers of the different lying-in hospitals in this and other countries.

The premature case, first to be noticed, occurred in a poor patient at the seventh month of utero-gestation. At first no cause could be assigned for the premature expulsion of the child, but, upon further inquiry, it was ascertained that the woman had been labouring under syphilis for some time, and was then under the influence of mercury.

In eight out of the fourteen protracted cases, the secale cornutum was exhibited in powder, in doses of from twenty-five grains to half a drachm; and in five the results were most satisfactory. One case deserved particular notice.

A lady was in labour of her fourth child. Upon entering the chamber, Mr. J. was informed that her former labours had been very protracted, for which reason she had not sent so early as she otherwise would have done. Upon making the usual examination, the os uteri was found scarcely dilated beyond the size of a shilling, but there was a capability in it of further dilatation; a distinction, Mr. J. observed, of great practical importance. The uterine power was inefficiently and feebly exerted, and in this state the patient had remained for the last five hours. Twenty-five grains of the ergot were then administered. In fifteen minutes there was a strong pain; the uterus then took on a regular and very effective action, and a fine healthy child was expelled within the hour. The recovery of the patient was more rapid and permanent than it had been after any of her previous labours.

Mr. J. then observed that, although he had experienced occasional disappointment in the administration of this medicine, he could not but entertain a most favorable opinion of it; and he thought a practitioner would be guilty of a dereliction of professional duty, if he went to a labour unprovided with it; as, in cases of hemorrhage, for instance, when it might sometimes be employed with great advantage, the delay occasioned in sending for it might be fatal to the patient. Besides, it was a part of the duty, also, of the accoucheur to relieve the sufferings of his patient, when it could be done upon sound obstetric principles; and he considered the ergot of rye as a most valuable acquisition to the means which might with great propriety be adopted in order to facilitate the parturient process. Mr. J. then exhibited a very neat and compact pocket case, made, according to his own directions, by Thomson, the surgeon's instrument maker, in Windmill street, containing three small stoppered bottles and a female catheter. One of the bottles, being graduated, is capable of holding three doses of the ergot; the other two are for tincture of opium and the carbonate of ammonia.

In the cases in which the feet presented, the children were lost from pressure on the funis, both being first labours.

In two of the breech cases, the children perished from the same cause.

The case of arm presentation was the most interesting. Mr. J. was called to the assistance of a pupil, and found the labour to be preternatural, the hand having dropped through the os externum, and the child in a state of putrescence. The tonic contraction of the uterus was so powerfully exerted as to preclude the possibility of turning, without almost the certainty of laceration. As no hope of the spontaneous evolution of the fœtus could be entertained, the alternate action of the uterus being altogether absent, Mr. J. determined on eviscerating the chest and abdomen of the child, and then to bring it away by fixing the crotchet in its pelvis or spine, in imitation of the spontaneous evolution, as recommended more particularly by Dr. Douglas and Dr. R. Lee; the latter of whom kindly assisted in the present instance. The arm of the child having been removed, the thorax was perforated and the ribs broken down, with the perforator and craniotomy forceps. The thoracic viscera were then removed, and subsequently those of the abdomen also. The crotchet having been firmly fixed in the pelvis of the child, the breech was brought down in a manner resembling the mechanism of the spontaneous evolution. The operation occupied about three quarters of an hour, and was accomplished without greater difficulty than might have been anticipated under such unfavorable circumstances. The woman recovered in the usual time, without the appearance of any morbid symptom.

In one of the cases of hemorrhage after delivery, the placenta was retained beyond the usual period, and flooding having ensued, half a drachm of the ergot was given, in addition to the means usually adopted; and in about twenty minutes the placenta was very easily removed, and the hemorrhage ceased altogether in about half an hour.

The forceps case was not worthy of particular notice. The patient had been in labour thirty-six hours, and, when Mr. J. was called in, the head of the child was low down in the pelvis, but the uterine action had subsided for nearly six hours. As the ergot was not at hand, and as some morbid symptoms began to be manifested, the forceps were employed, and the patient was delivered of a fine living child.

